# Risk factors of recurrent secondary hyperparathyroidism after adequate primary surgical treatment

**DOI:** 10.3389/fendo.2023.1063837

**Published:** 2023-02-03

**Authors:** Yu-Chi Kuo, Shang-Yu Wang, Yu-Liang Hung, Chih-Chieh Hsu, Hao-Wei Kou, Ming-Yang Chen, Chun-Yi Tsai, Chih-Hsiang Chang, Yu-Chao Wang, Jun-Te Hsu, Ta-Sen Yeh, Wei-Chen Lee, Chun-Nan Yeh

**Affiliations:** ^1^ Division of General Surgery, Department of Surgery, Linkou Chang Gung Memorial Hospital, Taoyuan City, Taiwan; ^2^ Graduate Institute of Clinical Medical Sciences, Chang Gung University, Taoyuan City, Taiwan; ^3^ College of Medicine, Chang Gung University, Taoyuan City, Taiwan; ^4^ Kidney Research Center, Department of Nephrology, Linkou Chang Gung Memorial Hospital, Taoyuan City, Taiwan

**Keywords:** secondary hyperparathyroidism, parathyroidectomy, end-stage renal disease, recurrence, parathyroid

## Abstract

**Background:**

Secondary hyperparathyroidism (SHPT) is a common condition in patients with end-stage renal disease (ESRD) who are on dialysis. Parathyroidectomy is a treatment for patients when medical therapy has failed. Recurrence may occur and is indicated for further surgery in the era of improved quality of care for ESRD patients.

**Methods:**

We identified, 1060 patients undergoing parathyroidectomy from January, 2011 to June, 2020. After excluding patients without regular check-up at our institute, primary hyperparathyroidism, or malignancy, 504 patients were enrolled. Sixty-two patients (12.3%, 62/504) were then excluded due to persistent SHPT even after the first parathyroidectomy. We aimed to identify risk factors for recurrent SHPT after the first surgery.

**Results:**

During the study period, 20% of patients who underwent parathyroidectomy at our institute (in, 2019) was due to recurrence after a previous parathyroidectomy. There were 442 patients eligible for analysis of recurrence after excluding patients with the persistent disease (n = 62). While 44 patients (9.95%) had recurrence, 398 patients did not. Significant risk factors for recurrent SHPT within 5 years after the first parathyroidectomy, including dialysis start time to first operation time < 3 years (*p* = 0.046), postoperative PTH >106.5 pg/mL (*p* < 0.001), and postoperative phosphorus> 5.9 mg/dL (*p* = 0.016), were identified by multivariate analysis.

**Conclusions:**

The starting time of dialysis to first operation time < 3 years in the patients with dialysis, postoperative PTH> 106.5 pg/mL, and postoperative phosphorus> 5.9 mg/dL tended to have a higher risk for recurrent SHPT within 5 years after primary treatment.

## Introduction

Secondary hyperparathyroidism (SHPT) is a condition specific to patients on dialysis with chronic renal insufficiency or end-stage renal disease (ESRD). With the initial disturbance of calcium and phosphorus balance, hyperplasia of the parathyroid gland occurs and persists during the following clinical course. Due to the adverse effects of SHPT, including vascular and tissue calcification, increased cardiovascular risk, mineral bone disease, and hyporesponsive anemia (1), treatments for SHPT are imperative. The treatment for SHPT includes both medical and surgical treatment. The medical treatments include modulation of the calcium and phosphorous balance by dietary intake and dialysis, active vitamin D compound use, phosphate binders, and calcimimetics, such as cinacalcet (2). Even with the recently increased use of calcimimetics, surgery still plays an important role in the treatment of SHPT. While calcimimetic has been reported to have clinical benefits in terms of normalization of electrolytes and lowering parathyroid hormone (PTH) (3, 4), its long-term survival benefit cannot be demonstrated by current evidence (5, 6). In addition, most medical treatments will be ineffective after a period of application, and approximately 15% of the patients on dialysis will need parathyroidectomy after 5–10 years due to refractoriness to medical treatment or intolerance to side effects related to calcimimetics (1, 5, 7). Therefore, parathyroidectomy are the last resort for effective long-term treatment.

Recently, published epidemiological data on Taiwanese ESRD patients revealed that the prevalence of dialysis cases increased from, 62381 to, 82154 during the survey period (2010 to, 2018) (8). In addition, the quality of care was improved due to the installation of a pre-ESRD care program (8). The increase in patients undergoing dialysis and improved quality of care, no doubt, will increase the number of patients who must suffer from SHPT. Although the prevalence of dialysis is high in Taiwan, the percentage of ESRD patients undergoing kidney transplantation is as low as 4.1%. It is much lower than that in the United States (29.3%), Singapore (18.3%), and Hong Kong (39%) (9–11). Without kidney transplantation, the pathogenesis of SHPT cannot be interrupted, and recurrence can be expected. Given the increased number of patients undergoing dialysis, improved quality of care, and low rate of kidney transplantation, an increasing number of repeated parathyroidectomy procedures can be anticipated in the near future. Therefore, we investigated whether any risk factor exists to predict recurrence after adequate primary surgery of parathyroidectomy for SHPT.

## Methods

We reviewed the operative records regarding parathyroid surgeries in our division, the Division of General Surgery of Chang Gung Memorial Hospital (CGMH), Linkou, according to relevant codes of surgical procedures. The Internal Review Board of CGMH approved the present study, and the reference number is, 202201157B0. We investigated all the surgical records from January, 2011 to June, 2020. All the records were reviewed by two surgical residents (Kuo YC and Hung YL) and verified by an attending surgeon (Wang SY). We included ESRD patients who not only underwent parathyroidectomy at our institute but who also regularly visited our nephrological clinic for clinical check-up. In the clinic of nephrologists, all the ESRD patients with SHPT were treated medically first. In our institute, patients were referred to the surgical clinic by nephrologists only due to medical treatment failure, and surgeons are not involved in the primary care of ESRD patients. Patients who underwent renal transplantation during the study period or underwent parathyroidectomy due to primary hyperparathyroidism or parathyroid malignancy were excluded from later analysis.

Since supernumerary (5 or more, 5% to 30%) and ectopic parathyroid glands (28% to 46%) are common in SHPT (12), we defined “adequate primary surgical treatment” as patients did not encounter persistent disease instead of surgical procedure they had undergone. Total parathyroidectomy may be an adequate treatment for patients with 4 glands but not for patients with 5 glands. Therefore, we excluded patients who suffered from persistent SHPT after primary surgical treatment, namely, the first surgery for SPTH. The definition of persistence was PTH>300 pg/mL during the first 6 months after primary surgical treatment (13). Only after excluding patients with the persistent disease, we can ensure that patients enrolled for further analysis were all subjects with adequate primary surgical treatment.

### Clinical information and outcome evaluation

Demographic data, including age, sex, American Society of Anesthesiologists Physical Status (ASA-PS) classification system, information regarding dialysis, laboratory data, and intraoperative findings, were all collected. The laboratory data included calcium, phosphorus, and PTH. The intraoperative findings included the number of identified parathyroid glands and the largest diameter of the largest gland of patients. The preoperative laboratory data were obtained by blood test when patients were admitted to the surgical ward of index hospitalization for surgery. The postoperative calcium, phosphorus, and PTH in the present study were data within 24 hours after surgery. The definition of recurrence was PTH > 600 pg/mL (14, 15) within 5 years after primary surgical treatment. Since we excluded patients undergoing renal transplantation, the trigger of SHPT, namely, the persistent status of ESRD, was not eliminated in our cohort, and recurrence decades after primary hyperparathyroidism can be expected. Therefore, we arbitrarily set recurrence within 5 years as a relatively undesirable outcome in the present study.

### Statistical analysis

Categorical variables are summarized as counts and percentages. Continuous variables are summarized as the mean with standard deviation (SD) or median with interquartile range (IQR). The chi-square test was applied for the difference in categorical variables. Student’s *t-test* was used for numeric data. The cut-off value of laboratory data, including calcium, phosphorus, and PTH, were obtained by ROC curve analysis. The risk factors were analyzed by a time-to-event model (Cox proportional hazard model). All analyses were conducted in R studio version 1.3.959 (R Foundation for Statistical Computing).

## Results

**Figure 1:**
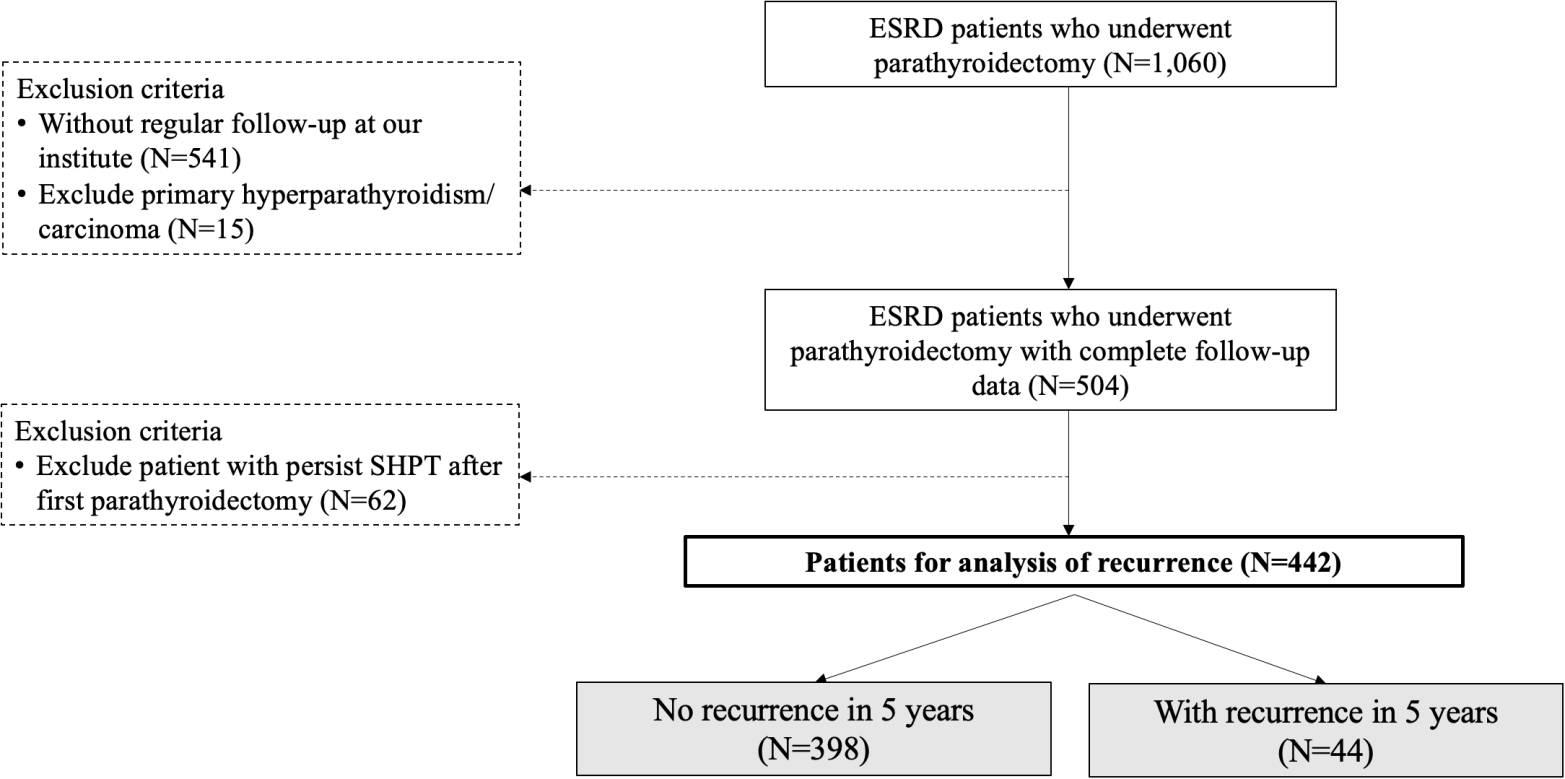
Flowchart for the stratification of the study cohort. ESRD, end-stage renal disease; SHPT, secondary hyperparathyroidism. The study period was January, 2011 to June, 2020.

**Figure 2:**
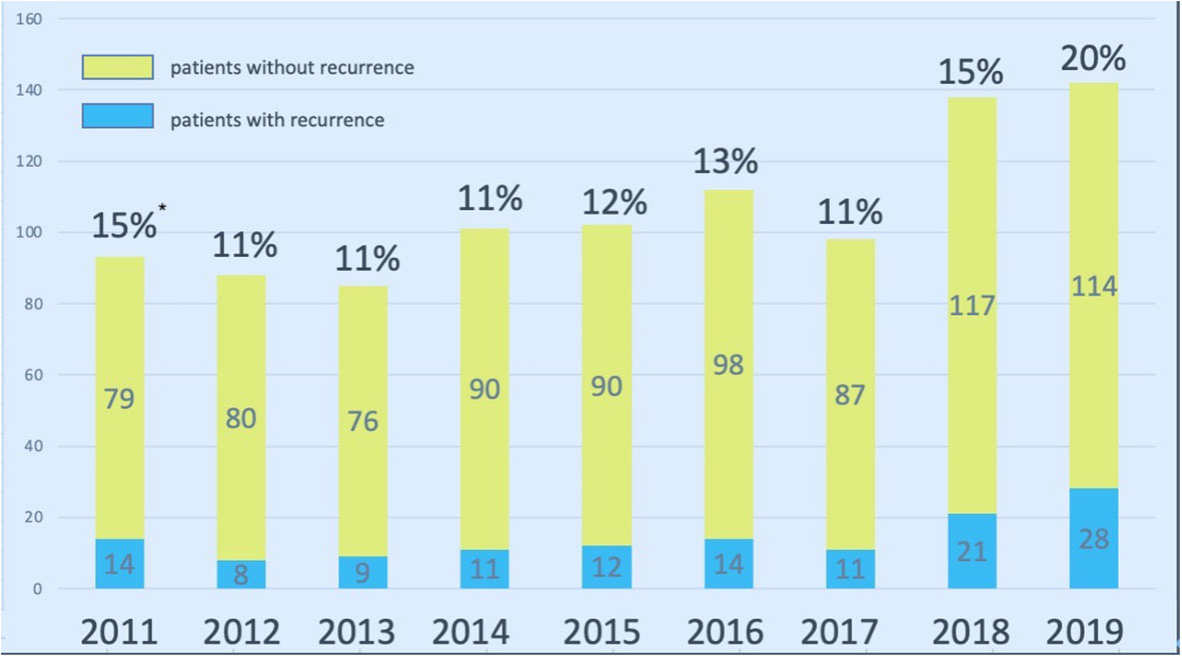
Surgical volume of parathyroidectomy for SHPT at Linkou Chang Gung Memorial Hospital from, 2011 to, 2019. * indicates the percentage of repeated parathyroidectomy for patients with recurrent SHPT. SHPT, secondary hyperparathyroidism.

During the study period, there were, 1060 patients undergoing parathyroidectomy for SHPT at our institute. After excluding patients without regular check-up at our institute, primary hyperparathyroidism, or malignancy of parathyroid glands, 504 patients underwent parathyroidectomy for SHPT and were regularly followed at the nephrological clinic at our institute (**Figure 1**). The number of patients who developed recurrent SHPT in, 2019 was as high as 20%, and the trend was slightly increasing (**Figure 2**). The rate of persistent SHPT after primary surgical treatment was 12.3% (64/504). The 5-year all-cause mortality was 8.79%.

In total, 442 patients completed the analysis after excluding the patients with the persistent disease (**Figure 1**). According to the aforementioned definition of recurrent SHPT, there were 44 patients (9.95%) with recurrence and 398 patients without recurrence (**Figure 1**). There was no significant difference between the patients with and without recurrence regarding the demographic data (**Table 1**), including age, sex, ASA-PS, and preoperative laboratory data (calcium, phosphorus, and PTH), except the duration from starting dialysis to primary surgical treatment (8.52 years vs. 10.44 years, *p* < 0.03).

**Table 1:** Demographic data of the patients for analysis of recurrence (N=442).

	No recurrence	Recurrence	*P*-value
Patients number(N)	398	44	
Age>65 years old (%)	64 (16.1)	5 (11.4)	0.55
ASA-PS score>=3	344 (86.4)	37 (84.1)	0.84
Male(%)	222 (55.8)	25 (56.8)	1
Dialysis start time to first operation (mean (SD))	10.44 (5.52)	8.52 (4.31)	0.03
Calcium level before operation (mg/dL)(mean (SD))*	10.64 (1.00)	10.55 (0.88)	0.6
Phosphorus level before operation (mg/dL)(mean (SD))*	5.69 (1.56)	5.74 (1.26)	0.82
PTH level before operation (pg/mL)(mean (SD))*	1472.71 (1015.62)	1351.65 (683.19)	0.44
PD history noted before(%)	108 (27.1)	15 (34.1)	0.42

ASA-PS, American Society of Anesthesiologists Physical Status; PTH, parathyroid hormone; PD, peritoneal dialysis.

**Figure 3:**
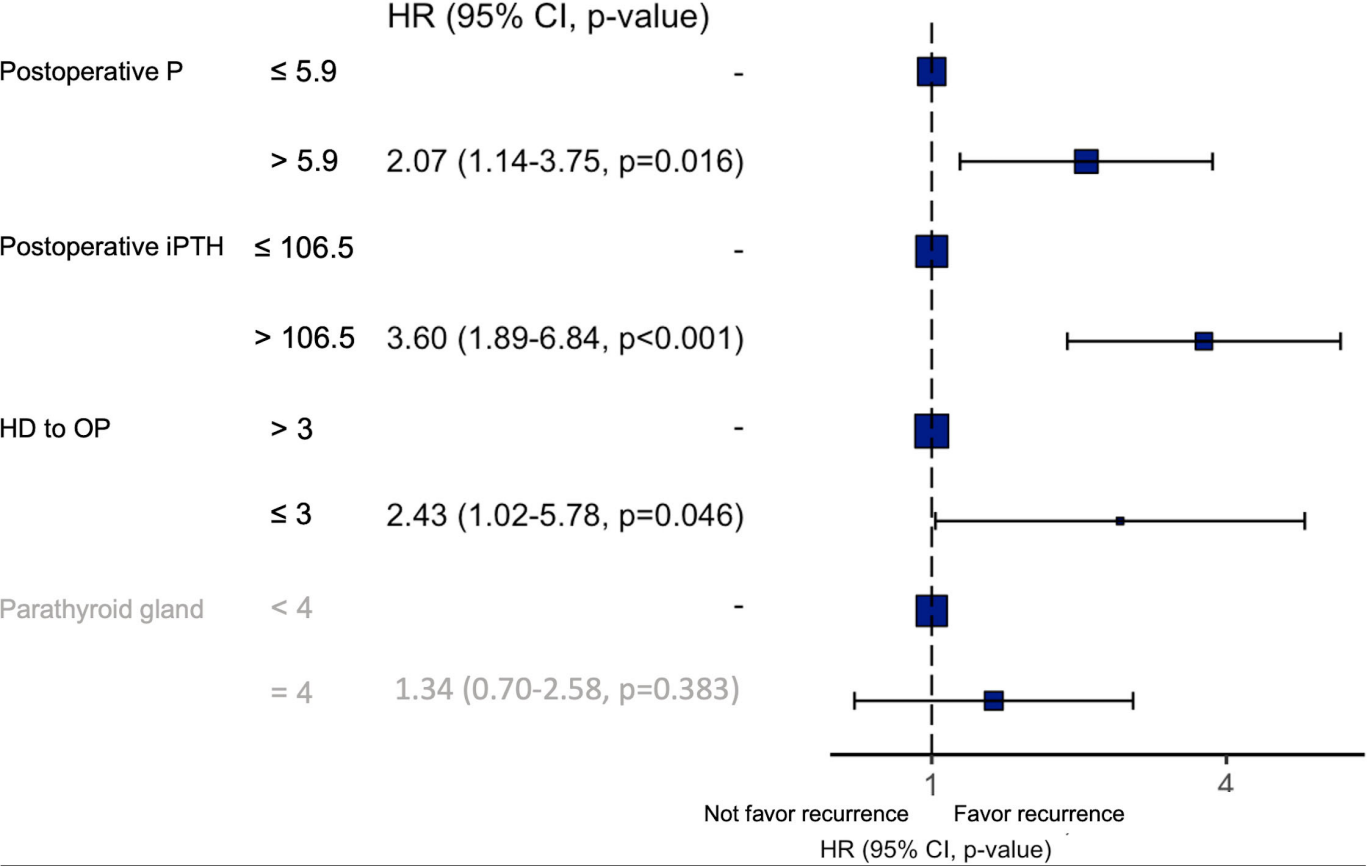
Results of the multivariate analysis of risk factors for recurrent SHPT. P, phosphorus; iPTH, intact parathyroid hormone; HD, hemodialysis; OP, operation; HR, hazard ratio.

After cut-off values of each of the variables were obtained *via* the ROC curve, risk factors for recurrence were analyzed. While dialysis starting time to first operation time less than 3 years, postoperative PTH over 106.5 pg/mL, postoperative operation over 5.9 mg/dL, and less than 4 glands identified were significant risk factors in the univariate analysis (**Table 2**), only the first 3 factors also demonstrated statistical significance in the multivariate analysis (**Figure 3**). Other investigated parameters, including age, sex, ASA, preoperative laboratory data, number of identified glands and size of glands, did not demonstrate statistical significance (**Table 2**).

**Table 2:** Univariate analysis of risk factors.

	HR	95% CI		
		Lower	Upper	*p*-value
Age>65	0.75	0.3	1.91	0.553
ASA-PS>3	0.97	0.43	2.18	0.947
Calcium level after operation>10(mg/dL)*	1.81	0.77	4.28	0.177
Calcium level before operation>11.3(mg/dL)*	1.04	0.51	2.11	0.912
male	1.06	0.58	1.92	0.858
Dialysis start time to first operation time <3 (years)*	0.42	0.18	0.99	0.048
PTH level after operation>106.5(pg/mL)*	3.82	2.10	6.94	<0.001
PTH level before operation>559.6 (pg/mL)*	1.14	0.35	3.67	0.831
Phosphorus level after operation>5.9(mg/dL)*	1.92	1.06	3.48	0.03
Phosphorus level before operation>4.9 (mg/dL)*	1.14	0.57	2.25	0.716
Not 4 parathyroid found	2.12	1.16	3.89	0.015
Parathyroid size>3.2(cm)*	1.48	0.46	4.77	0.515

*Cut-off were obtained by the ROC curve. ASA-PS: American Society of Anesthesiologists Physical Status; PTH: parathyroid hormone.

## Discussion

SHPT is an inevitable condition after long-term dialysis in patients with ESRD (1). An early small cohort study proved the clinical benefit of surgical treatment for SHPT (16) while a recent large cohort study also demonstrated its survival benefit (17). Parathyroidectomy is considered for patients with medically refractory SHPT. While calcimimetics reduced the need for parathyroidectomy in patients with SHPT, all-cause or cardiovascular mortality did not decrease (5, 6, 18). Therefore, parathyroidectomy currently still plays a role in the era of calcimimetics, especially for patients who are refractory to medical treatment (19).

The incidence of recurrent SHPT after parathyroidectomy has been reported and varies according to the definition of each study. Studies before, 2000 mainly focused on different surgical procedures and related outcomes. The number of patients in these reports seldom exceeded one hundred, and recurrent and persistent diseases were not clearly defined (20–22). The reported incidence of recurrence was 5% to 10% in the aforementioned studies. Studies with larger cohorts (range of patient numbers: 94 to 824), mainly conducted after, 2000, also reported recurrence of approximately 5% to 10% (23–27). However, those studies still lacked a clear definition of persistent or recurrent disease (23, 24, 26) and treated the necessity of reoperation as recurrence (27) or significant nonsurgical mortality during follow-up (25). Shih et al. conducted a study with clear definitions of both persistent disease and recurrence and demonstrated an overall recurrence rate of 11.7% (28). Our study excluded persistent disease, and the recurrence rate was 9.95%, similar to the currently reported data.

Most reports regarding recurrence mainly focused on comparisons between different surgical procedures (ex. subtotal parathyroidectomy and total parathyroidectomy with/without autotransplantation) or compared various perioperative adjuncts (ex. preoperative imaging technique or intraoperative PTH monitor); our study simply provided another point of view and investigated recurrence for patients after adequate surgical treatment or without persistent disease after primary surgical treatment. Regarding the choice of surgical procedures, published large retrospective studies or prospective randomized studies did not demonstrate the superiority of any specific procedures regarding surgical complications and recurrence (25, 27, 29). In our work, we defined “adequate primary surgical treatment” as patients who did not encounter persistent disease instead of surgical procedures they had undergone since the “true” total parathyroidectomy may be differently defined in patients with supernumerary glands. While missed orthotopic or ectopic glands and supernumeraries are major risk factors for persistent disease (13), the number of identified glands during the primary surgery may not be significant once an adequate treatment effect is achieved, namely, no persistent disease.

Three independent risk factors for recurrence were identified in the present study, including postoperative day 1 PTH level (> 106.5 pg/mL), time from dialysis start to primary parathyroidectomy (≤3 years), and postoperative day 1 phosphorus level (> 5.9 mg/dL). The postoperative PTH level may be a surrogate of residual parathyroid tissue. Since we enrolled patients without further renal transplantation, the pathogenetic factor of SHPT actually does exist even after parathyroidectomy. Therefore, recurrence may be inevitable; the curve of cumulative incidence demonstrated an increasing trend, which coincided with our result (**Supplementary Figure 1**), and more residual parathyroid tissue may predispose patients to recurrence.

The pathogenesis of SHPT from early medically controllable disease to refractory to medical treatment has been well studied (2). Initially, presenting with diffuse hyperplasia, parathyroid glands in SHPT would further grow to nodular hyperplasia with underexpression of the calcium-sensing receptors and vitamin D receptors, which then rendered poor response to medical treatment, namely, calcimimetics and vitamin D analogs, respectively (1). Tominaga et al. demonstrated that nodular hyperplasia was more progressively hyperplastic (30). Gasparri et al. later demonstrated that the highest risk of recurrence occurs in the nodular forms where the proliferative fraction exceeds 1.5%. Our study revealed that a time from dialysis start to primary parathyroidectomy less than 3 years, which is shorter than the reported natural course (1), was a risk factor for recurrence. This may imply that these patients possessed glands with a higher proliferation ability, causing SHPT to be refractory to medical treatment. Further study of the histopathology of these patients is indicated to confirm the pathophysiological significance or implication of a short time period from dialysis start to primary parathyroidectomy.

This retrospective cohort study had several limitations. First, we only enrolled patients who underwent surgery and regularly visited our clinic. This approach biased the actual incidence of recurrence since some patients with recurrent diseases might receive further treatment at our hospital. Second, we only included clinical factors for the investigation of recurrence. While several biomarkers and pathological characteristics have been identified, incorporating these factors for analysis may provide a better understanding of the risk of recurrence. Third, we identified “starting time of dialysis to fist operation time < 3 years” as a risk factor. Since surgeons did not participate in the primary care of ESRD patients and failure of medical treatment for SHPT was based on the judgment of nephrologists, there was no clear criteria for surgical referral. This imposed a risk of bias on the result of our work. Finally, one of the risk factors, phosphorus level > 5.9 mg/dL, was identified in our study. However, we cannot endow clinical significance to it. While hyperphosphatemia can perpetuate the progression of SHPT, we only used data from one spot, namely, a blood sample on postoperative day 1. Further relationships between serial phosphorus data and the risk of recurrence may be considered.

In conclusion, elevated postoperative PTH levels and a shorter time from dialysis start to primary parathyroidectomy were risk factors for recurrence. Based on our results, intraoperative PTH monitoring may be helpful in daily practice. Histopathological studies for patients with a short dialysis history may reveal other potential risk factors associated with the molecular mechanism contributing to recurrence.

## Data availability statement

The raw data supporting the conclusions of this article will be made available by the authors, without undue reservation.

## Ethics statement

The studies involving human participants were reviewed and approved by The Internal Review Board of CGMH approved the present study, and the reference number is, 202201157B0. Written informed consent for participation was not required for this study in accordance with the national legislation and the institutional requirements.

## Author contributions

Concept and study design: S-YW, C-NY, W-CL. Data collection: Y-CK, Y-LH, C-CH, H-WK, M-YC. Analysis: C-YT, C-HC, S-YW, Y-LH, J-TH. Manuscript writing: Y-CK, Y-CW, S-YW. Final approval and review: T-SY, W-CL, C-NY.

## Supplementary material

Supplementary Figure 1 Cumulative incidence of SHPT recurrence and death without SHPT recurrence in ESRD patients after primary parathyroidectomy, namely, the first-time parathyroidectomy. The incidence of death without SHPT recurrence within 5 years was 8.79%.
