# Supplementary figures and images for: Risk factors of recurrent secondary hyperparathyroidism after adequate primary surgical treatment
Source: Front Endocrinol (Lausanne). 2023 Feb 3;14:1063837. doi: 10.3389/fendo.2023.1063837 (PMC9936184; doi:10.3389/fendo.2023.1063837)

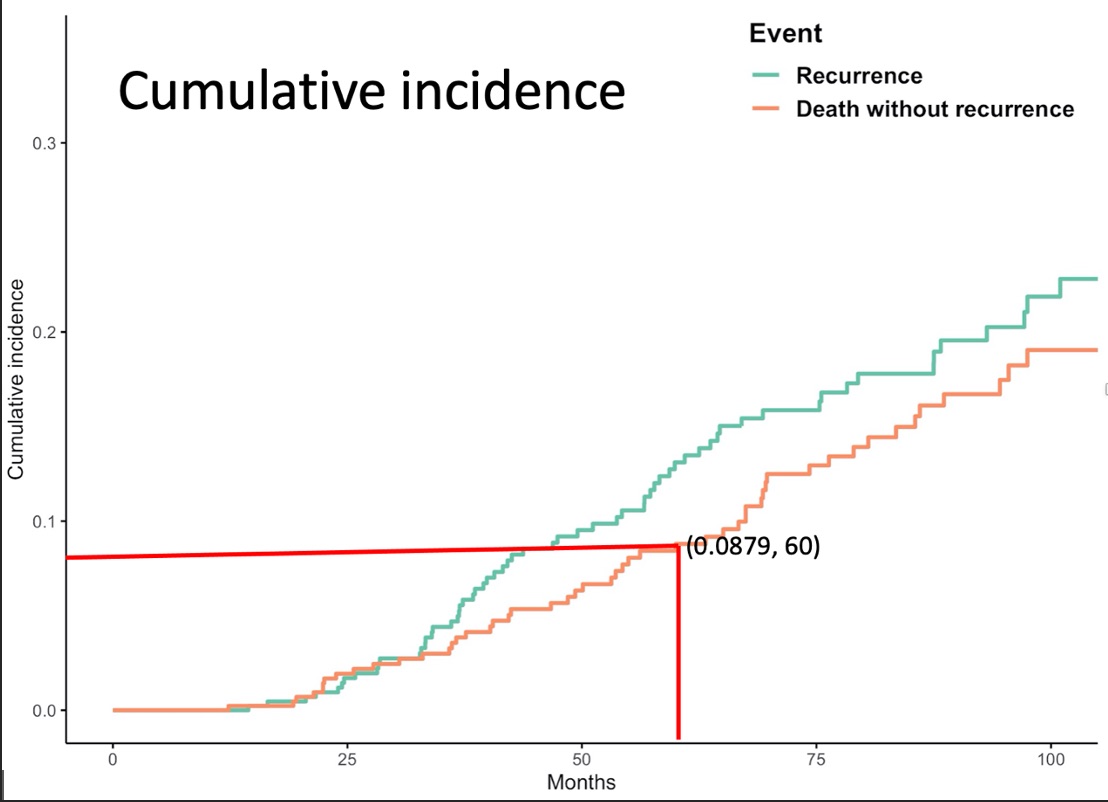

Supplement: Supplementary Figure 1 — Cumulative incidence of SHPT recurrence and death without SHPT recurrence in ESRD patients after primary parathyroidectomy, namely, the first-time parathyroidectomy. The incidence of death without SHPT recurrence within 5 years was 8.79%. [file Image_1.jpeg]
